# Elastase, α_1_-Proteinase Inhibitor, and Interleukin-8 in Children and Young Adults with End-Stage Kidney Disease Undergoing Continuous Ambulatory Peritoneal Dialysis

**DOI:** 10.1007/s00005-013-0265-7

**Published:** 2013-11-29

**Authors:** Bożena Polańska, Daria Augustyniak, Irena Makulska, Maria Niemczuk, Adam Jankowski, Danuta Zwolińska

**Affiliations:** 1 3rd Department and Clinics of Pediatrics, Immunology and Rheumatology of Developmental Age, Medical University, Koszarowa 5, 51-149 Wroclaw, Poland; 2 Department of Pediatric Nephrology, Medical University, Wroclaw, Poland; 3 Department of Pathogen Biology and Immunology, Institute of Genetics and Microbiology, University of Wroclaw, Wroclaw, Poland

**Keywords:** Neutrophil elastase, α_1_-Proteinase inhibitor, Interleukin-8, End-stage kidney diseases, Continuous ambulatory peritoneal dialysis

## Abstract

Peritoneal dialysis is one of the main modality of treatment in end-stage kidney diseases (ESKD) in children. In our previous work in chronic kidney disease patients, in pre-dialyzed period and on hemodialysis, the neutrophils were highly activated. The aim of this study was to assess an inflammatory condition and neutrophil activation in ESKD patients undergoing continuous ambulatory peritoneal dialysis (CAPD). Thirteen CAPD patients without infection, both sexes, aged 2.5–24 years, and group of healthy subjects (C) were studied. For comparative purposes the conservatively treated (CT) group of ESKD patients was included. Neutrophil elastase in complex with α_1_-proteinase inhibitor (NE-α_1_PI; ELISA), α_1_-proteinase inhibitor (α_1_PI; radial immunodiffusion) and interleukin-8 (IL-8; ELISA) were measured in the blood samples from CAPD, CT, and C group and in the peritoneal dialysate fluid (PDF) samples of patients on CAPD. A significantly increased plasma NE-α_1_PI levels (median 176.5 μg/L, range 85.2–373.2 μg/L; *p* < 0.00005), serum IL-8 (median 18.6 pg/mL, range 15.73–35.28 pg/mL; *p* < 0.05), and slightly decreased serum α_1_PI (median 1,540 mg/L, range 1,270–1,955; *p* ≤ 0.05) compared to the control groups were found. There were no significant differences of analyzed parameters between CAPD and CT patients. The concentration ratio of NE-α_1_PI, α_1_PI and IL-8 in blood/PDF was 29.97, 8.24, and 4.48, respectively. There were significantly positive correlations between serum and PDF concentration of α_1_PI and IL-8 (*r* = 0.613, *p* < 0.05; *r* = 0.59; *p* < 0.005, respectively). The results of our study demonstrate that neutrophils are highly activated in non-infected CAPD patients. The pivotal marker of this activation is NE-α_1_PI. It may contribute to chronic inflammation and tissues injury.

## Introduction

Uremia and long-term continuous ambulatory peritoneal dialysis (CAPD) may result in neutrophil disorders regarding their quantity, morphological or functional changes (Santamaria et al. 2009; Wasik et al. 1997). As a result, this leads to higher risk of developing inflammatory conditions, such as peritonitis and catheter-related infection (Stefaniak et al. 2002) as well as non-inflammatory complications including cardiovascular disease, fibrosis, impaired peritoneal ultrafiltration and an increase in mortality (Litwin et al. 2001; Yamamoto et al. 2010). Inflammation usually is associated with an overstimulation of neutrophils and release of their proteolytic enzymes, accompanied by the imbalance between cellular proteases and their inhibitors, and the activation of pro-inflammatory cytokines as well (Augustyniak et al. 2006; Korkmaz et al. 2005a; Lin and Huang 1994; Polańska et al. 2007). The hallmarks of neutrophilic inflammation are neutrophil elastase, α_1_-proteinase inhibitor (α_1_PI) and interleukin-8 (IL-8).

Free human neutrophilic elastase is regarded as one of the most potent proteolytic enzymes. It plays an important physiological function, being able to degrade foreign phagocytized particles both intracellularly (Shapiro 2002) and extracellularly (Papayannopoulos et al. 2010) and facilitate cell migration through vascular walls (Kaynar et al. 2008). One of the major and natural inhibitors of elastase is α_1_PI (Korkmaz et al. 2005a), which protects the surrounding tissues from enzyme-mediated destruction. Neutrophil elastase, both in the free form and in enzymatically, inactive complex with α_1_PI, may be a good indicator of the neutrophil activity (Polańska et al. 2004) as well as the degree of inflammatory reactions in acute and chronic diseases, including kidney disorders requiring dialysis (Donovan et al. 1993; Polańska et al. 2006, 2010; Shutov et al. 1999). IL-8 is the major chemotactic factor for neutrophils which also participates in their activation (Baggiolini and Clark-Lewis 1992) and degranulation (Segura et al. 1998). It also plays a significant role in the pathogenesis of inflammatory diseases (Baggiolini and Clark-Lewis 1992), cardiovascular diseases (Apostolakis et al. 2009) and fibrosis (Masunaga et al. 2003).

Although neutrophil elastase, α_1_PI and IL-8 play an important role in modulation of inflammation, the number of data referring to their significance in pediatrics patients with chronic kidney disease (CKD) is highly limited. Despite excessive data concerning the role of aforementioned mediators in kidney diseases analyzed separately, little is known about interrelations between them in pediatric patients in reference to renal replacement therapies. The goal of our study was to evaluate the blood and peritoneal dialysate fluid (PDF) concentrations of NE-α_1_PI, α_1_PI, and IL-8 in non-infected children and young adults with CKD on CAPD searching for differences between those parameters.

## Materials and Methods

### Subjects

The study covered 13 children and young adults with chronic and end-stage kidney disease (ESKD) on CAPD patients, aged 2.5–24 years (12.1 ± 6.8 years, mean ± SD), 7 females (54 %), and 6 males (46 %). The patients were dialyzed from 0.5 to 10 years (3.8 ± 3.6 years, mean ± SD) in the Department of Pediatric Nephrology in Wroclaw, Poland. All patients used conventional, low-glucose peritoneal dialysis solutions. Clinical causes which led to a CAPD therapy are shown in Table 1. All patients were clinically stable. In the time of investigation there were no clinical and conventional laboratory (C-reactive protein, erythrocyte sedimentation rate, total leukocytes count) signs of infection. None of the patients enrolled in the study had immunologic abnormalities. The patients with autoimmune disease had complete clinical and biochemical remission. Additionally, for comparative purposes the previously published conservatively treated (CT) group was included (Polańska et al. 2010). The CT group consisted of 13 patients, 6 females (46 %) and 7 males (54 %), aged 4–17 years (mean 12 ± 4.5 years), The causes of CKD in the CT group were: chronic glomerular nephritis (*n* = 4), hydronephrosis (*n* = 1), polycystic kidney disease (*n* = 4), kidney hypoplasia (*n* = 1), chronic interstitial nephritis (*n* = 1), and posterior urethral valves (*n* = 2). The control (C) group consisted of healthy subjects, both sexes, without chronic or recurrent diseases in anamnesis. Their results were regarded as normal values for NE-α_1_PI (group C1, *n* = 40, age range 1–16, mean 7 ± 4 years), for α_1_PI (group C2, *n* = 29, age range 1–24, mean 10 ± 7 years), and for IL-8 (group C3, *n* = 38, age range 1–24, mean 13.5 ± 7.5 years). None of the patients and controls received drugs having potential anti-inflammatory properties.

**Table 1:** Clinical features of patients on continuous ambulatory peritoneal dialysis suffering from chronic kidney disease and details of samples from patients

Subjects number	Age (years)	Sex	Chronic kidney disease causes	Peritoneal dialysis duration (years)	NE-α_1_PI (μg/L) blood/PDF	α_1_PI (mg/L) blood/PDF	IL-8 (pg/mL) blood/PDF
1	4	M	Polycystic kidney disease and hydronephrosis	4	259.08/3.1	2,060/370	12.83/2.64
2	5	M	Polycystic kidney disease	4	28.49/8.67	2,000/237	16.08/2.52
3	16	F	Chronic glomerulonephritis	10.5	176.5/2.3	1,540/207	18.6/27.85
4	24	M	Vesicoureteral reflux	10	537.5/9.9	169/166	124.15/14.73
5	10	M	Vesicoureteral reflux	1	85.2/0.8	1,430/201	32.43/6.76
6	24	F	Rheumatoid arthritis and amyloidosis	8	131.6/11.9	1,320/148	144.36/89.6
7	15	F	Neurogenic bladder	3.5	373.2/1.45	1,540/178	14.75/3.19
8	2.5	F	Congenital nephrotic syndrome	1.5	422.3/0.2	932/178	35.28/2.88
9	12	F	Polyvasculitis	0.5	76.5/18.2	–/166	16.03/3.25
10	11	F	Hydronephrosis and neurogenic bladder	0.5	125.5/–	1,120/–	25.83/–
11	12	F	Hemolytic-uremic syndrome	0.5	73.4/23.4	1,940/172	15.73/4.15
12	7	M	Primary hyperoxaluria	5	198.4/1.7	2,130/196	84.44/5.5
13	15	M	Vesicoureteral reflux	0.5	427.89/25	1,820/207	11.45/–

*F* female, *M* male, *PDF* peritoneal dialysate fluid, – not tested

### Material and Sampling Procedures

The material for investigation included peripheral venous blood which was obtained along with the blood drawn for routine laboratory tests from CAPD patients (B-CAPD), CT and healthy subjects, and included PDF from the abdominal cavity drawn simultaneously from CAPD patients (PDF-CAPD). Samples from PDF were obtained after 4 h dwell time. The blood samples, EDTA-treated blood samples and PDF were centrifuged (3,000 rpm, 10 min) within 2 h after collection. The serum, plasma and PDF were immediately divided into aliquots and stored at −80 °C until assayed.

This study was approved by the Research Ethics Committee of the Medical University of Wroclaw. The children were enrolled for the study with parental agreement. Informed consent was obtained from each patient’s parent and adult patients.

### Methods

Neutrophil elastase was determined by ELISA method in plasma and in undiluted PDF as a complex with its natural inhibitor, α_1_-proteinase using reagents manufactured by Merck (Darmstadt, Germany). The serum and undiluted PDF concentration of α_1_PI and IL-8/NAP-1 were investigated by radial immunodiffusion method using the Binding Site kit (Birmingham, UK), and ELISA kit of Bender MedSystems (Vienna, Austria), respectively. The analyses were performed according to the manufacturers’ recommendations. The limits of detection for NE-α_1_PI and IL-8/NAP-1 were <1.98 μg/L and <11 pg/mL, respectively. The coefficient of variation of α_1_PI repeat measurements was <5 %.

### Statistical Analysis

Statistical analysis was performed using the nonparametric the Mann–Whitney *U* test for independent variables. Spearman’s rank correlation coefficient was used to investigate any relationship between the parameters. The level of statistical significance was assumed to be *p* < 0.05. The analyses and illustrations were performed using StatSoft software (Statistica 8).

## Results

In all the analyzed samples of blood and PDFs investigated markers were detectable (Tables 1, 2). Significantly higher plasma NE-α_1_PI (*p* < 0.00005) and serum IL-8 (*p* < 0.05) levels were observed in CAPD patients in comparison to healthy subjects. In 4/13 cases (31 %) plasma NE-α_1_PI levels were within the normal range (60.78 ± 54.20 μg/L; mean ± 2SD) and in 5/13 (38 %) cases levels were above 200 μg/L. The median values of α_1_PI was slightly, but significantly (*p* ≤ 0.05) below the accepted norms. In the majority of samples [10/12 (83 %)] concentrations of α_1_PI were within the normal limits (1,862.01 ± 825.8 mg/L; mean ± 2SD). Particularly low concentrations of α_1_PI we found in the blood of two patients who had simultaneously elevated levels of NE-α_1_PI [first patient with vesicoureteral reflux (α_1_PI 169 mg/L, NE-α_1_PI 537.5 μg/L) and second with congenital nephrotic syndrome (α_1_PI 932 mg/L, NE-α_1_PI 422.3 μg/L)]. The analysis of α_1_PI in CAPD group depleted of at least one mentioned serum samples versus controls abolishes the indicated above significance. Serum IL-8 was above the reference range (17.41 ± 26.18 pg/mL, mean ± 2SD) in 3/13 (23 %) patients. One of them was the same person who had simultaneously elevated concentration of NE-α_1_PI and α_1_PI (with vesicoureteral reflux, IL-8 124.15 pg/mL, NE-α_1_PI 537.5 μg/L, α_1_PI 169 mg/L). There were no significant differences between B-CAPD and CT patients in reference to all three measured indicators (Table 2). The differences between CT patients and controls have been published previously (Polańska et al. 2010).

**Table 2:** Blood concentrations of the studied inflammatory markers in the CAPD, CT patients and healthy controls

Group	NE-α_1_PI (μg/L)	α_1_PI (mg/L)	IL-8 (pg/mL)
B-CAPD	176.5 (85.2–373.2)*	1,540 (1,270–1,955)**	18.6 (15.73–35.28)^#^
PDF-CAPD	5.89 (1.64–13.48)	187 (170.5–207)	4.15 (3.04–10.75)
CT	146.4 (118.8–183.1)^‡^	1,270 (1,070–1,650)	19.3 (12.6–153.7)
C1	59.38 (35.81–85.06)	–	–
C2	–	1,820 (1,590–2,130)	–
C3	–	–	13.49 (7.94–28.14)

Statistics was performed with Mann–Whitney *U* test, the results are presented as median value and interquartile range (25–75 %). Comparison B-CAPD and CT (for NE-α_1_PI, α_1_PI, and IL-8): not significant

*B-CAPD* blood of CAPD patients, *PDF-CAPD* peritoneal dialysate fluid of CAPD patients, *C1*, *C2*, *C3* healthy subjects for NE-α_1_PI, α_1_PI and IL-8, respectively

Comparison with normal value: * *p* < 0.00005, ** *p* ≤ 0.05, ^#^ *p* < 0.05, ^‡^ *p* < 0.000001

**Fig. 1:**
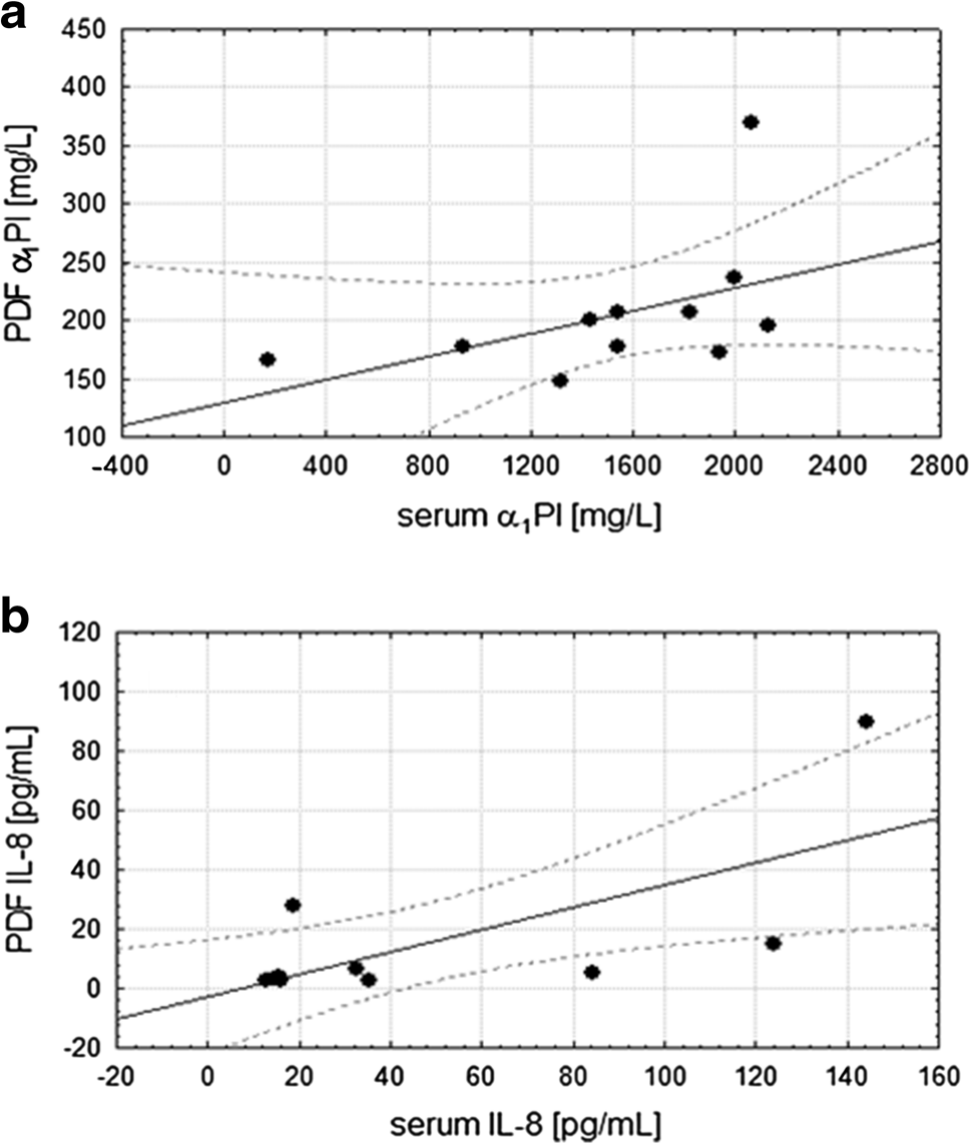
Correlations between serum and PDF α_1_PI concentrations (**a** *r* = 0.613, *p* < 0.05) and between serum and PDF IL-8 (**b** *r* = 0.59, *p* < 0.005) concentrations in patient with ESKD on CAPD

The B-CAPD levels of NE-α_1_PI were 30-fold higher, α_1_PI, eightfold higher whereas IL-8 only 4.5-fold higher comparing to their counterparts in PDF-CAPD. All tested inflammatory markers were in much lower concentrations in the PDF-CAPD compared to their levels accepted as the normative values in peripheral blood (NE-α_1_PI and α_1_PI about 10 times, IL-8 3.5 times lower). There were significant positive correlation between serum and PDF concentration of α_1_PI (*r* = 0.613, *p* < 0.05) and IL-8 *(r* = 0.59, *p* < 0.005), see Fig. 1. There were no correlations between the blood concentration of NE-α_1_PI, α_1_PI and IL-8 and between plasma and PDF concentrations of NE-α_1_PI. No statistically significant differences in concentrations of NE-α_1_PI, α_1_PI and IL-8 between the blood, PDF and duration of CAPD therapy were found.

Considering the potential influence of congenital anomalies of kidney and urinary tract (CAKUT) in CAPD patients on tested parameters, we did not find any significant differences between CAKUT group (Table 1: no. 1, 2, 4, 5, 10, 13) and that with other underlying diseases.

## Discussion

In long-therapy of CAPD patients with ESKD, the competent systemic and local, peritoneal immune defense mechanisms are very important. The results of our previous data clearly showed the high activity of neutrophils in young ESKD patients on hemodialysotherapy as well as in conservatively treated patients. It has been confirmed by considerable increase in circulatory level of NE-α_1_PI (Polańska et al. 2010). The results of our present study indicate that also in CAPD patients we found elevated levels of NE-α_1_PI in plasma. At the same time the concentration of NE-α_1_PI in the PDF were approximately tenfold lower in comparison to normal plasma levels, suggesting higher activity of neutrophils in peripheral blood than in the peritoneal cavity.

Due to the variety of relationships and interactions between the cells and their metabolites it is difficult to clearly identify the causes and the clinical consequences of increase activation of neutrophils in blood. The results of our study revealed that CAPD patients have a tendency to possess higher values of NE-α_1_PI and IL-8 than CT ones as compared to normal. We can only speculate that this is presumably in part a result of the body response to stimulating action of both the not fully removed circulated uremic toxins, invasiveness of the peritoneal dialysis procedure, inbiocompatibility dialysis solutions and IL-8 activities. Furthermore, it may be also connected with the property of neutrophil elastase in complex with α_1_PI to serve as a neutrophil chemoattractant (Banda et al. 1988). We can also assume that in the peritoneal cavity of our patients there was no acute inflammation which can over-stimulate neutrophils. The concentration of NE-α_1_PI and free elastase activity determined in the peritoneal cavity has already been studied in patients without peritonitis, showing the reduction of both form of enzyme (Donovan et al. 1993). Conversely, in peritonitis a common complication of CAPD in children, elevated levels of elastase both in plasma and dialysate fluid were observed (Shutov et al. 1999).

The consequences of observed NE-α_1_PI increase in pediatric patients for whom peritoneal dialysis is often a method of choice (especially in smaller children) may be diverse. Firstly, as the dynamic changes in the concentration of free neutrophil elastase or NE-α_1_PI in body fluids are very sensitive, its excessive amount may have significant importance for the preservation of homeostasis. Secondly, when elastase is released from overactivated neutrophils or after their apoptosis, unbounded neutrophil elastase can destroy extracellular matrix, basement membranes (Chua and Laurent 2006), and promote microvascular injury (Carden et al. 1998). This proteolytic feature of enzyme may prevent from fibrosis development, one of the most serious side effects of the long-term peritoneal dialysis (Yamamoto et al. 2010). On the other hand, however, neutrophil elastase paradoxically participates in excessive extracellular matrix deposition that leads to pulmonary fibrosis (Yamanouchi et al. 1998). Furthermore, neutrophil elastase inactivates complement components C3 and C5a, immunoglobulins, protease inhibitors, clotting factors and cell adhesion molecules that have a direct impact on the course of the inflammatory reactions (Lee and Downey 2001). All aforementioned autoaggressive properties of neutrophil elastase may contribute to the increase of the pathological remodeling of tissues in CKD patients. Thus, even a small amount of free neutrophil elastase present in the peritoneal cavity of our patients may support microinflammation of peritoneal membrane and ultrafiltration failure (Andreoli et al. 1994; Bos et al. 1991; Donovan et al. 1993). The reduced quantity/activity of neutrophil elastase may result also in neutrophil-related endothelial cell injury and increase vasculature permeability (Kaynar et al. 2008).

Neutrophil elastase activity is fully controlled by α_1_PI and other inhibitors as long as they are present in excess. It is worth to add that about 12 % of neutrophil elastase content in primary neutrophil granules is mobilized to the cell membrane where neutrophil elastase is resistant to inhibitors and remains catalytically active (Korkmaz et al. 2005b; Owen et al. 1995). The results of our study have shown that elevated concentrations of NE-α_1_PI were not accompanied by the α_1_PI increase in peripheral blood in both CKD groups. We suspect that this may be only an apparent shortage as a result of the rapid consumption of inhibitor molecules by excessively released neutrophil elastase and subsequent elimination of NE-α_1_PI complexes (Korkmaz et al. 2005a). Because α_1_PI is considered as a positive marker of acute inflammatory reaction, its low quantity probably reflects the lack of such a response in our patients. Another explanation may be the proteolytic degradation of α_1_PI by myeloperoxidase (Honda et al. 2009) or neutrophil elastase (Cantin et al. 1989), indirectly confirmed by the lack of significant correlations between NE-α_1_PI and α_1_PI both in the blood and PDF CAPD samples. Finally, α_1_PI inactivation may be the result of excessive oxidative stress which occurs in patients on dialysis (Zwolińska et al. 2009) followed by the release of free reactive oxygen species (Carp and Janoff 1980).

Identified relatively low concentrations of IL-8 in the serum of CAPD patients, although significantly higher when compared to healthy control (*p* = 0.036) indicate rather a chronic inflammation or microinflammation than the acute condition. This can be however a real risk for cardiovascular complications (Apostolakis et al. 2009). It is worth noting that in our patients, without clinical manifestations of peritonitis, it was the smallest differences in the concentrations of IL-8 between the serum and the PDF in comparison to NE-α_1_PI and α_1_PI. This may be partly the result of the easier compensation of pro-inflammatory cytokines by anti-inflammatory cytokines observed in children (Pereira 1995) or suppressive action of free radicals on the production of IL-8 (DeForge et al. 1992). In accordance with other data we speculate therefore that the source of IL-8 found in the PDF samples may be rather cytokine-activated peritoneal macrophages or fibroblasts (Lin and Huang 1994; Witowski et al. 2001) than neutrophils.

The lack of significant correlation found between IL-8 and NE-α_1_PI in the blood and PDF can provide that neutrophil degranulation followed by the release of elastase was also influenced by other factors, such as (not determined in this work) the components of complement, leukotrienes, and tumor necrosis factor α. Positive correlation between serum and PDF IL-8 and α_1_PI concentration, found in investigated samples, may be partly the consequence of the chronic or microinflammation presence in long-term dialysed uraemic patients, even without clinical signs. As demonstrated by Bergin et al. (2010) α_1_PI may play also an anti-inflammatory role by inhibiting neutrophil chemotaxis. It forms a complex with IL-8, and controls the binding of IL-8 with CXCR1 (neutrophil chemokine receptor). The interaction between neutrophil elastase, inhibitor α_1_-proteinase and IL-8 contributes to the perpetuating circle that facilitates the recruitment of inflammatory cells and amplifies the neutrophil-associated tissue destruction.

There are some limitations to the present study, thus all conclusions must be assessed carefully. First, issue is the low number of pediatric patients, reflecting the general difficulty in performing clinical studies in this population. Secondly, few of our patients have underlying inflammatory diseases which might affect the evaluated parameters. Thirdly, we did not evaluate the functional activities of the study parameters or other multidirectional cellular and molecular interactions influencing them. We did not perform also laboratory data excluding the primary α_1_PI deficiency. Finally, because we performed a clinical study, our conclusions are based rather on associations and cannot prove causality.

In conclusion, the results of our study due to the fact of increased concentration of NE-α_1_PI in peripheral blood and their presence in PDF demonstrate that neutrophils are highly active in non-infected CAPD patients. The investigation of the innate immunity components might show new indices on pathogenesis, identify novel therapeutic targets for treatment, and prevent from many complications improving the life quality of these patients.

## Abbreviations

CAKUT: Congenital anomalies of kidney and urinary tract
CAPD: Continuous ambulatory peritoneal dialysis
CT: Conservative treatment
CKD: Chronic kidney disease
ESKD: End-stage kidney disease
PDF: Peritoneal dialysate fluid
B-CAPD: Blood-CAPD
α_1_PIα_1_: α_1_-Proteinase inhibitor
NE-α_1_PI: Neutrophil elastase complexed with α_1_-proteinase inhibitor
IL-8: Interleukin-8
ELISA: Enzyme-linked immunosorbent assay
